# Baseline urate level and renal function predict outcomes of urate-lowering therapy using low doses of febuxostat and benzbromarone: a prospective, randomized controlled study in a Chinese primary gout cohort

**DOI:** 10.1186/s13075-019-1976-x

**Published:** 2019-09-02

**Authors:** Nan Liang, Mingshu Sun, Ruixia Sun, Ting Xu, Lingling Cui, Can Wang, Lidan Ma, Xiaoyu Cheng, Xiaomei Xue, Wenyan Sun, Xuan Yuan, Hui Zhang, Hailong Li, Yuwei He, Aichang Ji, Xinjiang Wu, Changgui Li

**Affiliations:** 1 The Department of Endocrinology and Metabolism, The Affiliated Hospital of Qingdao University, Qingdao, China; 2 Qingdao University, Qingdao, China; 3 Shandong Provincial Key Laboratory of Metabolic Diseases and Qingdao Key Laboratory of Gout, the Affiliated Hospital of Qingdao University, Qingdao, China; 4 Department of Rheumatology and Clinical Immunology, the Affiliated Hospital of Qingdao University, Qingdao, China; 5 Department of Geratology, the 971th Hospital of PLA, Qingdao, China; 6 Institute of Metabolic Diseases, Qingdao University, Qingdao, China

**Keywords:** Gout, Benzbromarone, Febuxostat, Urate-lowering treatment

## Abstract

**Background:**

Low doses of febuxostat or benzbromarone are widely used in Asian countries, but lacking studies to compare the efficacy and safety of the two urate-lowering drugs.

**Methods:**

To compare the efficacy and safety of low-dose febuxostat with low-dose benzbromarone in patients with primary gout, a randomized controlled, open-label trial was performed among male patients with primary gout for urate-lowering therapy (ULT) in a dedicated gout clinic in China. Randomization was carried out by a third-party institution according to random number table. Patients were randomly assigned 1:1 to febuxostat group (Feb group) (20 mg daily) or benzbromarone group (Ben group) (25 mg daily) and treated for 12 weeks. General information and biochemical data were collected at baseline and at every visit monthly. Clinical characteristics before and after the ULT were analyzed in the two groups by SPSS and EmpowerStats software.

**Results:**

Two hundred forty patients were enrolled and randomized in the two groups, with 214 patients completing 12 weeks’ ULT (105 in the Feb group and 109 in the Ben group). After 12 weeks, substantial percentages of patients in both Feb and Ben group achieved the target serum uric acid (sUA) (< 360 μmol/L) and serum urate levels were reduced significantly for both groups (Feb 39.5% and 156.83 μmol/L vs. Ben 35.7% and 163.99 μmol/L). Multivariate analysis suggests baseline sUA level and renal function were associated with the outcome of the rate of achieving target sUA (RAT). Sub-group analysis suggests low doses of febuxostat and benzbromarone rendered better RAT for patients with sUA < 540 μmol/L and creatinine clearance rate (Ccr) ≤ 110 mL min^−1^ 1.73 m^−2^ at baseline. The drugs were well tolerated, and the incidence of gout flares in Feb group was similar with that in Ben group (22.85% vs. 33.94%).

**Conclusion:**

Overall, febuxostat 20 mg daily and benzbromarone 25 mg daily reduced sUA, and gout patients with sUA level < 540 μmol/L or Ccr ≤ 110 mL min^−1^ 1.73 m^−2^ at baseline had better chance to achieve target uric acid levels. The current study suggests sUA level and renal function are key factors to consider when recommending low doses of febuxostat and benzbromarone to gout patients.

**Trial registration:**

Registered with ChiCTR, No. ChiCTR1800019352 (retrospectively registered).

## Background

Gout has been the most common inflammatory arthritis worldwide [1], which is characterized by the formation and deposition of monosodium urate crystals in joints and the consequent inflammation or erosion around the victim joints [2]. Patients with gout often present comorbid conditions such as hypertension, diabetes mellitus, hyperlipidemia, and chronic kidney disease (CKD). Hyperuricemia is the prelude of all these conditions, including gout [3–7]. The management of hyperuricemia thus plays a key role in the treatment of gout and, potentially, of its comorbidities. A series of guidelines recommend target serum uric acid (sUA) levels of urate-lowering therapy (ULT) are 360 μmol/L (6 mg/L) in moderate gout and 300 μmol/L (5 mg/L) in severe gout [8–15].

For the sake of preventing second gout attack induced by unstable sUA levels and avoiding unwanted side effects of high dose ULT drugs, “start low go slow” strategy with low-dose initiation of ULT drugs and gentle dosage escalation is recommended in ULT [13–16]. Particularly, we focused on two drugs here: xanthine oxidase inhibitor febuxostat and uricosuric agent benzbromarone. In the case of febuxostat, FDA approves 40 mg and 80 mg, which are widely adopted in the guidelines of American College of Rheumatology and European League Against Rheumatism. But 20 mg, even 10 mg, is widely used in clinical practice in particular in Asian countries [17]. Benzbromarone was approved for use in Europe and Asia, but was not licensed in the USA. In contrast to Europe or British guidelines, most Asian consensus or guidelines recommend benzbromarone as first-line ULT and 50 mg is the most common recommended starting dose [13, 15, 16]. However, 25 mg as starting dose is common in clinical practice of gout management in Asia and was recommended in Chinese expert consensus [13]. So far, a number of studies have shown “start low go slow” dosing strategies can achieve satisfactory rates of achieving targets among Asian patients for both febuxostat and benzbromarone [18–20]. However, clinical studies observing the effectiveness and safety of low-dose febuxostat or benzbromarone in ULT of the primary gout patients are lacking.

Overall, the current study aimed to address the following: (1) general effectiveness and safety of low-dose febuxostat (20 mg) and benzbromarone (25 mg) in Chinese primary gouty patients and (2) explore the clinical parameters which may influence the rate of achieving target sUA (RAT) or adverse events when applying low doses of febuxostat and benzbromarone. Therefore, we performed this randomized controlled, open-label study to compare febuxostat (20 mg/day) with benzbromarone (25 mg/day) on the efficacy and safety in ULT in a Chinese primary gout cohort. We hypothesized that this study might provide clues in choosing the right drug, at a right dosage, for right patients.

## Patients and methods

### Patients enrollment

We screened patients who visited the dedicated Gout Clinic of the Affiliated Hospital of Qingdao University. The male patients who had diagnosed with primary gout based on the 2015 ACR/EULAR criteria [21] with fasting uric acid ≥ 420 μmol/L were enrolled. The patients were excluded for any of the following reasons: having a recent gout attack or taking any urate-lowering drugs or other medicine affecting the serum uric acid in the past 2 weeks before enrollment, presenting abnormally high levels of transaminase (> 2.0 times of the upper normal limit) or serum creatinine (> 1.0 time of the upper normal limit), and suffering from urinary calculi, rheumatoid arthritis, or other serious associated conditions. The study was approved by the ethics committee of Affiliated Hospital of Qingdao University, and informed consents were obtained from all patients. This study was registered with ChiCTR, No. ChiCTR1800019352.

### Study design

**Fig. 1:**
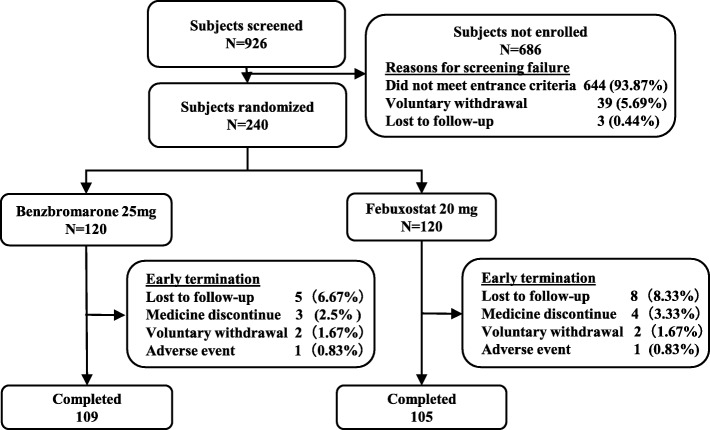
Flow chart of the study

This was a prospective, single-center, randomized controlled, open-label study (Fig. 1). The patients were randomized 1:1 according to random number table into the febuxostat group (Feb group), in which patients were treated with febuxostat 20 mg/day + sodium bicarbonate 3 g/day, or benzbromarone group (Ben group), with benzbromarone 25 mg/day + sodium bicarbonate 3 g/day. Patients took fixed dose of febuxostat or benzbromarone daily in the morning and took 1 g of sodium bicarbonate three times a day for 12 weeks and were followed at the end of 4, 8, and 12 weeks.

Baseline information was collected before the initiation of treatment, after a washout period of 14 days. In the washout period, patients discontinued the use of ULT drugs. If gout flare occurs, the patients would be treated with colchicine and NSAIDs and the washout period would be restarted.

The following data were obtained at baseline: age, gender, disease history, height, body weight, waist circumference, blood pressure, serum alanine aminotransferase (ALT), serum aspartate aminotransferase (AST), serum glucose (GLU), serum triglyceride (TG), serum cholesterol (TC), serum creatinine (Cr), and serum uric acid (UA). The biochemistry tests were repeatedly measured at every visit. The creatinine clearance rate (Ccr) were used to assess the renal function (Cockcroft-Gault (CG) equation, Ccr = (140-age) × body weight (kg)/0.818 × Scr (μmol/L)). Patients who interrupted to take drugs for more than 3 days and who failed to be followed up were judged as withdrawn cases.

Colchicine and/or etoricoxib were administered at any sign of gout flares during the trial. Every gout flare was recorded at the next follow-up visit. Hepatic protectants diammonium glycyrrhizinate, silibinin, or polyene phosphatidyl choline were prescribed at physicians’ discretion in cases whose serum transaminases exceeded the upper limit of normal by 2-fold.

The primary endpoints of this study included the rates to achieve ULT target (sUA < 360 μmol/L) and the decreasing ranges of sUA at the end of treatment. The secondary endpoint was the number of gout flares during the treatment and the side effects.

### Data analysis

The per-protocol analysis was performed with SPSS 22.0 and EmpowerStats software. Data was presented as mean ± SD or proportions. Comparisons of baseline data between the two groups were performed using the *t* test or Mann-Whitney rank-sum test for measurement data and chi-squared test for categorical data. Logistic regression analysis was used to identify factors predicting the sUA target-achieving. To access the variation tendency of repeatedly measured data of two groups, data was analyzed with mixed model for repeated data by EmpowerStats software. The computing methods of this mixed model emphasized the comparison of the changing trend of variables between the two groups, which was the changing trend of uric acid and other indexes in this case.

## Results

### Baseline characteristics of the patients

From November 2015 to September 2017, 240 patients were randomly assigned and given either the Feb group or Ben group, and 214 patients completed 12 weeks’ ULT (105 in the Feb group and 109 in the Ben group). The information on recruitment is shown in Fig. 1. There were two withdraw cases (one in each group) who stopped drugs because of liver function damage (> 3 times of upper normal limit and cannot be reverted by taking routine liver-protective drugs only). The major baseline demographic and gout-associated clinical characteristics (sUA, Ccr) are well-balanced between the two groups (Table 1).

**Table 1:** Baseline characteristics of the subjects

	Febuxostat *n* = 105	Benzbromarone *n* = 109	*P* value
Age, years	52.42 ± 11.73	50.27 ± 14.15	0.23
Onset age, years	41.80 ± 10.57	43.19 ± 13.15	0.48
Height, cm	172.65 ± 5.65	172.39 ± 5.83	0.77
Body weight, kg	78.89 ± 9.49	77.27 ± 9.55	0.28
BMI, kg/m^**2**^	26.59 ± 3.03	26.10 ± 2.75	0.25
Waistline, cm	94.60 ± 7.41	92.31 ± 7.92	0.10
Waist-to-hip ratio	0.92 ± 0.05	0.92 ± 0.04	0.65
Systolic BP, mmHg	128.42 ± 13.55	126.78 ± 16.93	0.51
Diastolic BP, mmHg	83.47 ± 8.74	82.07 ± 9.67	0.32
Coexisting conditions			
Hypertension	25 (22.9)	20 (19.0)	0.33
Cardiovascular disease	5 (4.6)	7 (6.7)	0.60
Diabetes	7 (6.4)	3 (2.9)	0.30
Hyperlipidemia	32 (29.4)	34 (32.4)	0.91
Fatty liver	26 (23.9)	28 (26.7)	0.88
Tophus	26 (23.9)	21 (20)	0.33
Family history of gout	24 (22.0)	26 (24.8)	0.86

Data given as mean ± SD or number (%). *P ≤ 0.05. **P ≤ 0.01. *BMI* body mass index, *BP* blood pressure

### Logistic regression analysis of characteristics related to target-achieving

**Fig. 2:**
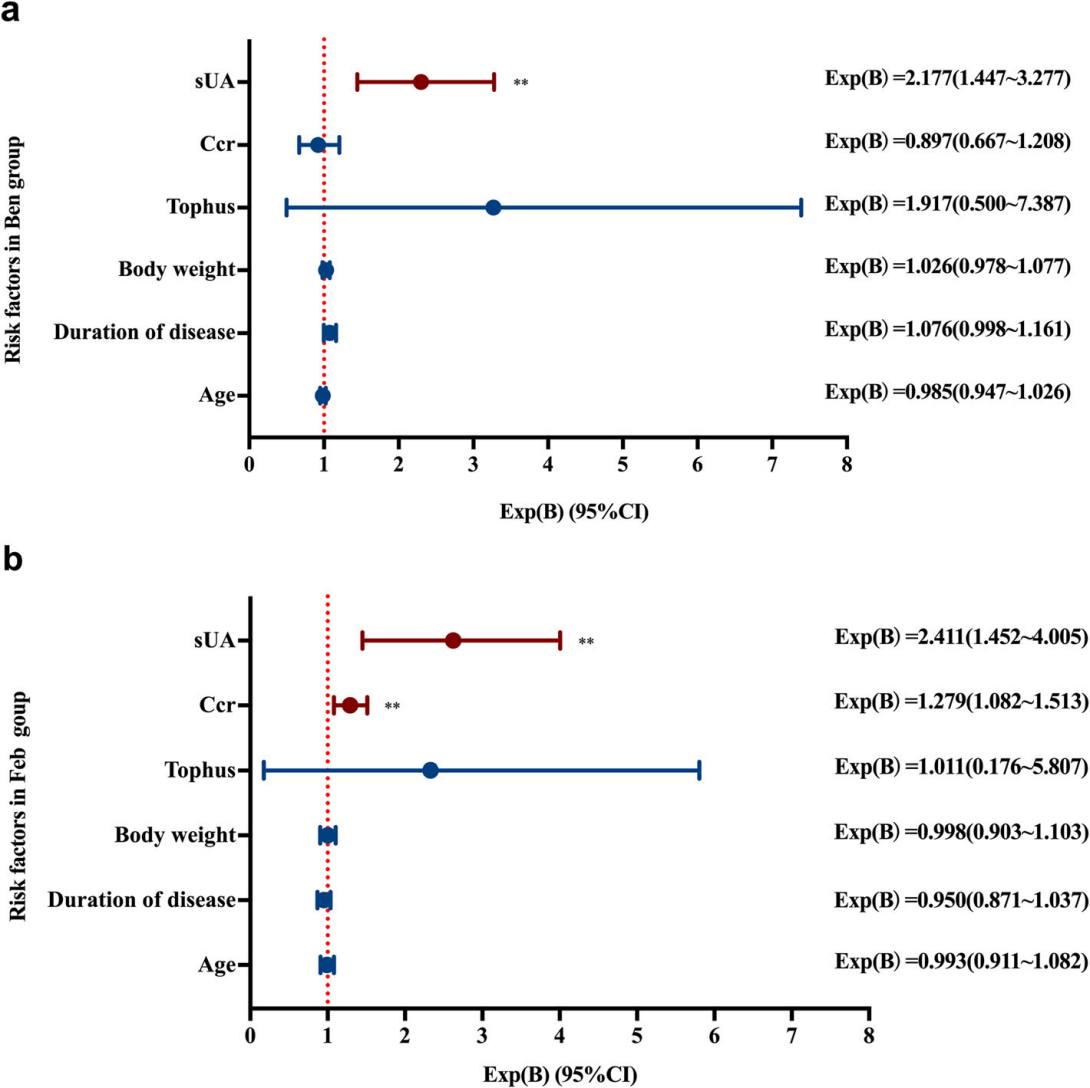
Clinical factors associated with RAT. **a** Clinical risk factors associated with RAT in the Ben group. **b** Clinical factors associated with RAT in the Feb group. The data was analyzed by logistic regression multivariate analysis. Exp(B) = the exponent of b. The Exp(B) values indicated the risk to fail to achieve treatment target for each unit additional increase on the covariate. The “each unit” was 60 μmol /L in sUA, 10 mL min^−1^ 1.73 m^−2^ in Ccr, present (1) or absent (0) in tophus, 1 kg in body weight, 1 year in duration of disease and age. **P < 0.01

According to the results of the *t* tests and chi-squared tests of the baseline variables between patients who achieved or did not achieve the treating targets, age, body weight, duration of disease, and presence or absence of tophus, baseline sUA and Ccr were brought into logistic regression analysis to explore risk factors that impacted target achieving rates. The baseline levels of sUA (Exp(B) = 2.411, 95% CI 1.452–4.005) and Ccr (Exp(B) = 1.279, 95% CI 1.082–1.513) were regarded as independent risk factors to predict achieving the ULT target in the Feb group. And the sUA was the only factor in the Ben group (Exp(B) = 2.177, 95% CI 1.447–3.277) (Fig. 2).

### The RAT and major disease-related biochemical parameters after ULT

As the target of ULT was set as the endpoint sUA level < 360 μmol/L, we calculated the number and proportion of people who achieved that target. The overall RAT of all the patients was 37.9% (81/214). 38.1% (40/105) of patients in the Feb group and 37.6% (41/109) in the Ben group reached the goal. There were no significant differences between the two groups (*P* > 0.05). Low dose of febuxostat and benzbromarone led to decreased levels of sUA throughout the treatment period which dropped from 561.10 ± 71.63 (μmol/L) to 404.27 ± 81.10 (μmol/L) in the Feb group and from 554.90 ± 66.73 (μmol/L) to 390.91 ± 92.87 (μmol/L) in the Ben group, respectively. Average uric acid levels significantly decreased in both the Feb Group and Ben Group (*P* < 0.001) (Table 2).

**Table 2:** Major clinical parameters during the trial

	Baseline	4 weeks	8 weeks	12 weeks
sUA, μmol/L				
Febuxostat	561.10 ± 71.63	405.43 ± 78.20^##^	429.41 ± 94.71^##^	404.27 ± 81.10^##^
Benzbromarone	554.90 ± 66.73	381.58 ± 92.8^##^	400.57 ± 79.91^##^	390.91 ± 92.87^##^
CCr, mL/min				
Febuxostat	105.93 ± 30.61	108.17 ± 32.14	108.61 ± 30.73	110.87 ± 30.93^##,^*
Benzbromarone	108.86 ± 29.12	108.25 ± 27.27	108.15 ± 28.41	108.08 ± 28.78
Cr, μmol/L				
Febuxostat	84.21 ± 15.10	82.39 ± 14.65#	83.38 ± 14.55	80.83 ± 15.13^##,^*
Benzbromarone	81.13 ± 12.19	81.63 ± 12.03	79.83 ± 9.82	82.14 ± 12.48
BUN, mmol/L				
Febuxostat	5.56 ± 1.21*	5.66 ± 1.19	5.69 ± 1.39	5.59 ± 1.45
Benzbromarone	5.02 ± 1.40	4.84 ± 1.33	5.00 ± 1.36	5.83 ± 7.90
TG, mmol/L				
Febuxostat	1.70 ± 0.65	1.98 ± 0.85^#,^**	2.03 ± 1.15^#,^*	1.97 ± 0.89*
Benzbromarone	1.93 ± 0.93	1.81 ± 0.89	1.95 ± 1.04	1.87 ± 0.85
TC, mmol/L				
Febuxostat	4.95 ± 0.93	4.94 ± 0.95	4.89 ± 0.88	5.02 ± 0.87
Benzbromarone	5.21 ± 0.98	5.12 ± 0.91	5.27 ± 0.92	5.20 ± 0.88
ALT, U/L				
Febuxostat	32.45 ± 15.96	43.77 ± 22.10^##,^**	42.73 ± 24.13^##,^**	42.88 ± 24.20^##,^*
Benzbromarone	27.74 ± 13.87	29.96 ± 19.74	28.05 ± 15.18	30.08 ± 20.94
AST, U/L				
Febuxostat	22.90 ± 11.38	26.97 ± 11.22^##,^*	25.365 ± 7.88^##^	27.32 ± 14.59^##,^**
Benzbromarone	22.16 ± 6.87	22.88 ± 9.76	22.21 ± 7.63	22.75 ± 8.64
GLU, mmol/L				
Febuxostat	5.76 ± 0.57	5.63 ± 0.60	5.73 ± 0.66	5.74 ± 0.75
Benzbromarone	5.76 ± 0.93	5.67 ± 1.03	5.65 ± 0.59	5.80 ± 0.94

Data given as mean ± SEM. *P ≤ 0.05, **P ≤ 0.01, Feb group vs. Ben group. ^#^*P* ≤ 0.05, ^##^*P* ≤ 0.01, before vs. after treatment

The average levels of liver or renal function-related parameters were within normal range throughout the trial except the ALT level in the Feb group slightly exceeded the upper limit of normal range. Ccr, TG, ALT, and AST of the Feb group increased during the ULT compared to the baseline levels (Table 2). Twenty-five-milligram benzbromarone treatment did not alter the major liver, lipid metabolic, and renal function-related parameters (Table 2).

### Subgroup analysis based on baseline sUA and renal function

**Fig. 3:**
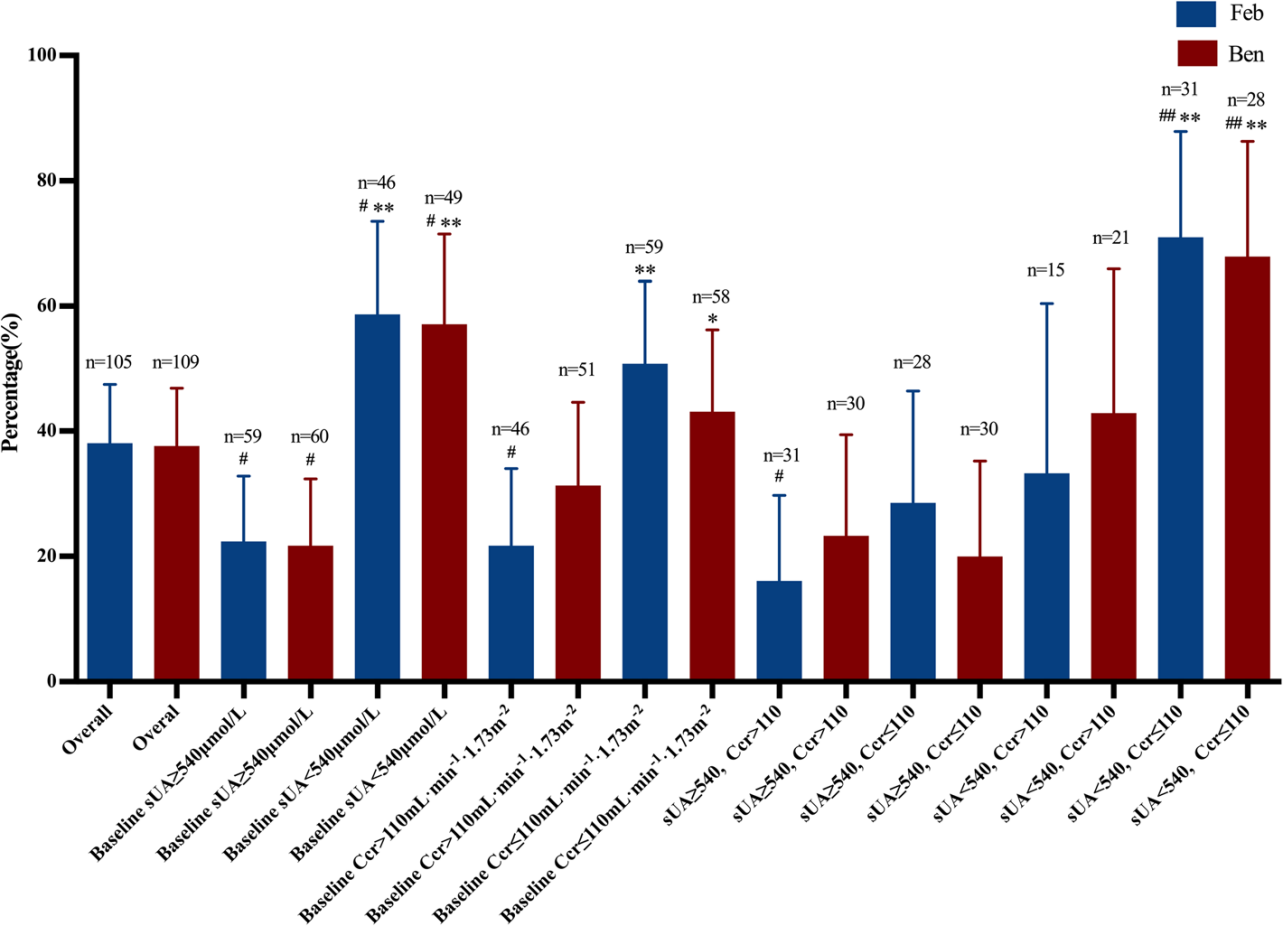
Subgroup analysis of RAT based on the baseline sUA level and renal function. The RAT in the Feb group, the Ben group, and subgroups after ULT for 12 weeks. The detail date was in the text. *P < 0.05 and **P < 0.01 vs. RAT of the alternative subgroup within the Feb or Ben group; ^#^*P* < 0.05 and ^##^*P* < 0.01 vs. overall RAT of the Feb or Ben group

Given baseline sUA and renal function associated with RAT outcome suggested in multivariate analysis, we performed a subgroup analysis of RAT based on baseline sUA level (< or ≥ 540 μmol/L) and renal function (Ccr ≤ or > 110 mL min^−1^ 1.73 m^−2^). Gout patients in the Feb group and Ben group with baseline sUA < 540 μmol/L achieved higher RATs (Feb 58.7% and Ben 57.1%) after 12-week treatment than the overall patients (Feb 38.1% and Ben 37.6%). On the other hand, patients with baseline sUA ≥ 540 μmol/L reached lower RATs (Feb 22.4% and Ben 21.7%) than the overall patients (Fig. 3). When considering baseline renal functions, gout patients with Ccr > 110 mL min^− 1^ 1.73 m^− 2^ exhibited lower RATs for the Feb group (21.7%) compared to the RATs of overall patients (38.1%) (Fig. 3). The difference was not significant for the Ben group (31.4% vs. 43.1%). However, RATs of gout patients with Ccr > 110 mL min^−1^ 1.73 m^−2^ (Feb 21.7%, Ben 31.4%) are much lower than the RATs of patients with Ccr ≤ 110 mL min^−1^ 1.73 m^−2^ (Feb 50.8%, Ben 43.1%).

Then, we further stratified patients into 4 subgroups in the Feb or Ben group by combining baseline sUA level and renal function. We were able to demonstrate that RATs of gout patients with baseline sUA < 540 μmol/L and Ccr ≤ 110 mL min^−1^ 1.73 m^−2^ increased to over 60% (71% for the Feb group and 67.9% for the Ben group). The RATs for the Feb group (16.1%) or Ben group (23.3%) were both low in the subgroups with baseline sUA ≥ 540 μmol/L and Ccr > 110 mL min^−1^ 1.73 m^−2^. Otherwise, RATs of the other two subgroups are not superior to the overall RATs despite one baseline levels of sUA or Ccr factor exists (Fig. 3).

### Safety evaluation

During the 12 weeks of the study, the Ben group showed similar occurrence rate of gout flares with the Feb group (33.94% vs 22.85%, *P* > 0.05) (Table 3). There was no significant difference between the two groups in the occurrence rate of more than one flare (10.09% vs 6.66%, *P* > 0.05). The overall occurrence of gout flair was 28.50% (Table 3).

**Table 3:** Adverse events

	Febuxostat *N* = 105	Benzbromarone *N* = 109	Total *N* = 214
Gout flare	24 (22.85)	37 (33.94)	61 (28.50)
Once	17 (16.19)	26 (23.85)	43(20.09)
Twice	5 (4.76)	11 (10.09)	16 (7.48)
More than twice	2 (1.90)	0	2(0.93)
Transaminase elevation from normal	35(33.33)	26 (23.85)	61 (28.50)
1~2 × ULN	25 (23.81)	22 (20.18)	47 (21.96)
2~3 × ULN	9 (8.57)	3 (2.75)	12 (5.61)
> 3 × ULN	1 (0.95)	1 (0.92)	2(0.93)
New-onset CKD based on CCr	0	4 (3.67)	4 (1.87)
CCr: 50–80 mL min^−1^ 1.73 m^−2^	0	4 (3.67)	4 (1.87)
CCr: < 50 mL min^−1^ 1.73 m	0	0	0
Cardiovascular events	0	0	0
Skin reaction	0	0	0
Other adverse events	0	0	0

Data presents the number of patients (percentage). *ULN* upper limit of normal

The treatment-related AEs were similar between the Feb group and the Ben group; most of them were mild to moderate extent (Table 3). The patients with elevated transaminase to over 40 U/L were 35/105 in the Feb group and 26/109 in the Ben group; most of them were mild (elevated to 40–80 U/L) to moderate (elevated to 80–120 U/L) in intensity. Among those patients, 10 in the Feb group and 4 in the Ben group were of clinical significance and in need to take hepatic protectants (transaminase > 2 upper limit of normal (ULN)). One in each group was suggested to withdraw from the study because they needed to stop ULT. All the hepatic events were resolved after hepatic treatment or stopping ULT.

The CKD stages were used to evaluate the safety of low-dose urate-lowering drugs (Table 3). During the 12-week treatment, four patients in the Ben group and none in the Feb group developed CKD, all in the stage I, and the differences of the safety indexes were not statistically significant between the two groups.

The skin reactions (drug eruption, exfoliation, dermatitis medicamentosa) were not found in the patients. None of the major adverse cardiac events (recurrent angina pectoris, nonfatal myocardial infarction, cardiac failure, malignant arrhythmia, death from cardiovascular causes) nor any other adverse event was found.

## Discussion

To our knowledge, this is the first head-to-head study comparing the efficacy and safety of low-dose urate-lowering agents, febuxostat and benzbromarone, in primary gout patients. Febuxostat is an XO inhibitor and metabolized in the liver and excreted mainly by the urinary system and intestinal tract. Benzbromarone is a uricosuric agent which inhibits urate transporter 1 (URAT1) that is located to the brush border membrane of renal proximal tubular cells to reduce the reabsorption of uric acid [22, 23]. In Europe and the USA, febuxostat is widely recommended as second-line ULT drug, and benzbromarone is not available in the USA and not available in most Europe countries due to a rare occurring hepatoxicity [24, 25]. However, both febuxostat and benzbromarone are recommended by most Chinese or Japan clinical guidelines as first-line ULT drugs [13–15]. Given start low go slow ULT strategy is widely accepted in gout management, the current study provided novel clinical evidence to support the notion that low-dose ULT may bring satisfactory clinical outcome to certain gout patients with baseline sUA and renal function at certain ranges.

In this randomized controlled study, low-dose febuxostat (20 mg/d) reduced sUA by approximately 157 μmol/L, and the overall sUA < 360 μmol/L target-achieving rate was 38.1%. This result is strongly consistent with the previous study conducted by Naoyuki, in which febuxostat was also used with 20 mg/day in a Japanese gout cohort [26]. The result is also similar to other studies, of which the RATs varied from 22.5 to 52.4% in Chinese, Japanese, or western gout cohorts using febuxostat 40 mg/day [27–29]. It was poorly reported for the urate-lowering efficacy of benzbromarone. Our study shows low-dose benzbromarone (25 mg/day) reduced sUA by averagely 164 μmol/L, and the RAT was 37.6%, an outcome comparable to that of 20 mg/day febuxostat. The result of stratification analysis based on the baseline sUA level and Ccr is exciting and of great value in dose selection. Our data indicates that both drugs work effectively in a low dose for most Chinese gout patients with sUA level < 540 μmol/L. As for most patients with sUA level ≥ 540 μmol/L, a dose escalation may be needed to catch the sUA targets. Most patients (67.9% in the Ben group and 71% in the Feb group) with both sUA < 540 μmol/L and Ccr ≤ 110 mL min^−1^ 1.73 m^−2^ can achieve the treating goal with a low dose of ULT drugs, and the percentage of patients did not markedly change from the 4th week to the 12th week.

The incidence of gout flare was 22.85% in the febuxostat group. A recent study conducted on Japanese population found the incidence was higher (36.0% vs 20.8%, *P* = 0.048) in patients treated with febuxostat 40 mg/day than 10 mg/day initiation for 12 weeks [18]. Another study also found that the incidence of gout flare was higher in the febuxostat 40 mg group than in the 20 mg group (17.6% vs 5.7%, *P* < 0.05) [26]. These results indicate that the recurrence rate of gout flare tends to be initial-dose-dependent.

Gout is often accompanied by nephropathy and metabolism-related diseases such as diabetes and dyslipidemia. The ideal urate-lowering agent is supposed to improve the comorbidities or at least not to aggravate them. Theoretically, febuxostat is more suitable for patients with urate overproduction, and benzbromarone is for patients with urate underexcretion. Situations are usually more complicated in actual practice when comorbidities or combined medicines exist. In safety evaluation, neither of the two groups manifested severe drug-related side effects, and the average blood glucose did not change markedly in neither group. Febuxostat’s hepatoxicity is generally tolerable according the previous studies [30–32]. We did observe transaminase levels in the Feb group increased mildly comparing with baseline and the Ben group. However, there was no statistical difference in the proportion of patients with elevated transaminase between the two groups, and transaminase elevation in the Feb group was generally mild to moderate and self-limited (23.81% increased by 1–2ULN, 8.57% increased by 2–3ULN). The TG level elevated in the Feb group, which was also consistent with the previous study. It is better choosing febuxostat with a second thought for those with hypertriglyceridemia. In contrast, the average levels of transaminase and TG did not change in the Ben group. Benzbromarone has been reported to be related to several cases of hepatic failure and was withdrawn from the market in some European countries in 2003 [24, 25], which was not observed in our study using low-dose benzbromarone.

The Ccr level increased (approximately 5 mL/min) after 12 weeks’ treatment in the Feb group but not in the Ben group, while the sUA decreased comparably between the two groups. These results imply that febuxostat but not benzbromarone may provide extra renal protection irrespectively of urate lowering. One recent randomized trial also found that febuxostat delays the progression of renal dysfunction in Japanese patients [33].

Kidney function is associated with hyperuricemia, and it will make a difference in the urate-lowering treatment response. The sUA target-achieving rate was higher in patients with Ccr ≤ 110 mL min^−1^ 1.73 m^−2^ than in patients with Ccr>110 mL min^−1^ 1.73 m^− 2^ (in the Feb group, 50.8% vs 21.7%, *P* < 0.01; in the Ben group, 43.1%vs 31.4%, *P* < 0.01), especially in the febuxostat group. The impact of Ccr on the target-achieving rate may have resulted from its role on urate excretion. The kidney is the main organ of urate excretion, and the urate excretion fraction is determined by filtration in the glomerular and reabsorption and secretion in the tubular. In early stage of gouty nephropathy, nocturia and hyposthenuria are observed, accompanied by increased urinary urate excretion, indicating impaired renal tubular function and reduced water and uric acid reabsorption [34, 35].Thus, reduced uric acid reabsorption at early stage of gouty nephropathy may favor urate lowering effect of ULT drugs. Given the distinct ULT mechanisms of febuxostat and benzbromarone, febuxostat likely benefits more from change of uric acid resorption since benzbromarone also targets uric acid reabsorption and a competitive effect may exist.

The limitations of the study include its open-label design and relatively short experimental period (12 weeks). A dose-escalation design with longer period would provide more information to guide using these two ULT drugs in real-world clinical practice. However, RCT design and participating patients with reasonably large size warrant the robustness of the results and derived conclusions. In addition, the study population was with relatively normal renal function, so the conclusion may not apply in gout patients with impaired renal function.

## Conclusion

In conclusion, both febuxostat 20 mg/day and benzbromarone 25 mg/day can significantly reduce sUA levels with minimal adverse effects, and a substantial percentage of gout patients can achieve RAT, particularly for patients with baseline sUA < 540 μmol/L and Ccr≦110 mL min^−1^ 1.73 m^−2^. The results strengthened the concept of start low go slow in ULT and provided evidence to guide febuxostat and benzbromarone use to achieve optimal clinical outcome among gout patients.

## Data Availability

The datasets used and analyzed during the current study are available from the corresponding author on reasonable request.

## Abbreviations

ALT: Alanine aminotransferase
AST: Aspartate aminotransferase
Ccr: Creatinine clearance rate
CKD: Chronic kidney disease
Cr: Creatinine
FEUA: Fraction excretion of uric acid
GLU: Serum glucose
RAT: Rate of achieving target sUA
sUA: Serum uric acid
TC: Cholesterol
TG: Triglyceride
ULT: Urate-lowering therapy
